# Metabolic responses to high *p*CO_2_ conditions at a CO_2_ vent site in juveniles of a marine isopod species assemblage

**DOI:** 10.1007/s00227-016-2984-x

**Published:** 2016-09-20

**Authors:** Lucy M. Turner, Elena Ricevuto, Alexia Massa Gallucci, Maurizio Lorenti, Maria-Cristina Gambi, Piero Calosi

**Affiliations:** 1 Marine Biology and Ecology Research Centre, Plymouth University, Drake Circus, Plymouth, PL4 8AA UK; 2 Department of Marine Sciences, University of Gothenburg, Box 460, 405 30 Göteborg, Sweden; 3 Stazione Zoologica Anton Dohrn, Department of Integrative Marine Ecology, Villa Dohrn - Benthic Ecology Centre, 80121 Ischia, Naples Italy; 4 Département de Biologie, Chimie et Géographie, Université du Québec à Rimouski, Rimouski, QC G5L 3A1 Canada

## Abstract

We are starting to understand the relationship between metabolic rate responses and species’ ability to respond to exposure to high *p*CO_2_. However, most of our knowledge has come from investigations of single species. The examination of metabolic responses of closely related species with differing distributions around natural elevated CO_2_ areas may be useful to inform our understanding of their adaptive significance. Furthermore, little is known about the physiological responses of marine invertebrate juveniles to high *p*CO_2_, despite the fact they are known to be sensitive to other stressors, often acting as bottlenecks for future species success. We conducted an in situ transplant experiment using juveniles of isopods found living inside and around a high *p*CO_2_ vent (Ischia, Italy): the CO_2_ ‘tolerant’ *Dynamene bifida* and ‘sensitive’ *Cymodoce truncata* and *Dynamene torelliae*. This allowed us to test for any generality of the hypothesis that *p*CO_2_ sensitive marine invertebrates may be those that experience trade-offs between energy metabolism and cellular homoeostasis under high *p*CO_2_ conditions. Both sensitive species were able to maintain their energy metabolism under high *p*CO_2_ conditions, but in *C. truncata* this may occur at the expense of [carbonic anhydrase], confirming our hypothesis. By comparison, the tolerant *D. bifida* appeared metabolically well adapted to high *p*CO_2_, being able to upregulate ATP production without recourse to anaerobiosis. These isopods are important keystone species; however, given they differ in their metabolic responses to future *p*CO_2_, shifts in the structure of the marine ecosystems they inhabit may be expected under future ocean acidification conditions.

**Electronic supplementary material:**

The online version of this article (doi:10.1007/s00227-016-2984-x) contains supplementary material, which is available to authorized users.

## Introduction

As a result of the ongoing anthropogenic increase in CO_2_ emissions, the level of seawater *p*CO_2_ is expected to increase, resulting in a decrease in oceanic pH by 0.4–0.5 pH units by the year 2100 (Caldeira and Wickett 2003; IPCC 2014); a phenomenon known as ocean acidification (OA). However, despite a plethora of recent studies, we still know relatively little about the homoeostatic metabolic responses of marine ectotherms to high *p*CO_2_ conditions compared to other climate-driven parameters such as temperature (Somero 2005; Pörtner 2008; Gunderson and Stillman 2015; c.f. Stillman and Paganini 2015). Most previous investigations have been single species orientated and taxonomically restricted, mainly to heavily calcified marine invertebrate species (e.g. corals, sea urchins and molluscs), which have been predicted to experience the greatest negative impacts from any decrease in oceanic pH due to calcium carbonate dissolution, being adversely affected by changes in ocean chemistry (Fabry et al. 2008; Wittmann and Pörtner 2013). The fact that shell biomineralisation processes would likely need to be upregulated in low pH conditions can arguably have a significant impact on an organism’s energy metabolism, with changes predicted to occur in the direction and the intensity of the metabolic response (e.g. Pörtner 2008; Lannig et al. 2010; Melatunan et al. 2011; Maas et al. 2012; Ivanina et al. 2013; Melatunan et al. 2013; Klok et al. 2014). Such changes would likely lead to a trade-off between an organism’s capacity to maintain or increase its metabolic rates and other fundamental, energy demanding, physiological processes, such as growth and reproduction. This would ultimately be most detrimental to an organism’s overall fitness (Saba et al. 2012; Edmunds et al. 2013; Rivest and Hofmann 2014; Uthicke et al. 2014).

In comparison, crustaceans, including juvenile and larval stages have been shown to be relatively tolerant to decreases in seawater pH predicted to occur for the end of the century (Widdicombe and Spicer 2008; Melzner et al. 2009; Ries et al. 2009; Ross et al. 2011; Whiteley 2011; Branch et al. 2012; Arnberg et al. 2013; c.f. Walther et al. 2011; Small et al. 2015). This is probably due to the fact that many crustacean species are characterised by fairly low degrees of calcification, but are also able to successfully regulate internal acid–base balance, and whilst this necessitates an increase in metabolic effort, overall homoeostasis appears to remain intact (Spicer et al. 2007; Small et al. 2010; Rastrick et al. 2014). However, within crustaceans, a number of groups remain understudied in terms of the effect of *p*CO_2_ levels projected to occur by the end of the century (Caldeira and Wickett 2003; IPCC 2014) on the metabolic costs of maintaining homoeostasis. One such group is the marine isopods which are considered to be important keystone species in marine ecosystems, including in shallow water seagrass habitats (see review by Poore and Bruce 2012). The isopod cuticle contains magnesium calcite, which is highly soluble (Andersson et al. 2008; Neues and Epple 2008), and as a result isopods are potentially at risk of being negatively affected by OA conditions (Alenius and Munguia 2012; Jakubowska et al. 2013; Munguia and Alenius 2013; Wood et al. 2014). Furthermore, very few studies examining the effects of OA on fundamental physiological processes have focussed on juvenile (post-larval) stages of lower calcifying marine invertebrates (i.e. those with lighter, less mineralised skeletons), with only a handful of studies published to date (e.g. Carter et al. 2013; Ceballos-Osuna et al. 2013; Knapp et al. 2015; Small et al. 2015; Zheng et al. 2015), despite juveniles being considered as sensitive bottlenecks for population recruitment, including in crustaceans (Walther 2010; Small 2013). Instead, where impacts of OA on early life stages have been investigated, the vast majority of these studies have been restricted to measurements of shell dissolution rates and/or ecological and life history traits such as growth, survivorship and/or settlement in heavily calcified species (Melzner et al. 2009; Byrne 2011; Ross et al. 2011; Whiteley 2011). It is therefore evident that, overall, juveniles have been overlooked, partly as a result of often being seen as identical, but simply morphometrically smaller versions of adult species (Page 2009). During the juvenile phase, many significant physiological and behaviour changes take place, including metamorphosis and organogenesis, onset of sexual maturity and changes in diet and habitat (Hadfield 2000; Byrne 2011; Ross et al. 2011). Consequently, recent work has suggested that juveniles may, in fact, be particularly physiologically sensitive, therefore acting as bottlenecks for the longevity of individual species, especially under ongoing global change scenarios (Walther et al. 2010; Byrne 2011; Walther et al. 2011). It is surprising therefore that quantifying the physiological response to OA in juveniles of marine invertebrates has not attracted further interest.

Shallow water high CO_2_ vents have been used as analogues to investigate the potential ecological and evolutionary implications of OA (e.g. Hall-Spencer et al. 2008; Kroeker et al. 2011; Rodolfo-Metalpa et al. 2011; Calosi et al. 2013a, b; Lucey et al. 2015; Ricevuto et al. 2015; Gambi et al. 2016). At the high *p*CO_2_ vent of the Castello Aragonese at Ischia (Italy), the invertebrate fauna have been characterised along the existing CO_2_ gradient. This includes assemblages comprising juveniles of several isopod species with different distribution patterns, seemingly as a result of their tolerance or sensitivity to high *p*CO_2_ conditions (Kroeker et al. 2011; Ricevuto et al. 2012). Based on their distribution, marine invertebrates found inside and outside these naturally acidified areas have been defined as either ‘tolerant’ (abundant inside and often outside the low pH/high *p*CO_2_ areas) or ‘sensitive’ (found only outside the vents in similar habitat) (Calosi et al. 2013b; Turner et al. 2015). Recent work on an annelid assemblage at this site has shown clear differences between the metabolic responses of closely related species that overlap in their distribution patterns. Those species classed as ‘tolerant’ are able to maintain their metabolic rate levels during acute exposure to elevated *p*CO_2_, whereas those classed as ‘sensitive’ are not, and, instead show significant up or downregulation of metabolic rate (Calosi et al. 2013b). To some extent, these patterns of relationships between species’ distribution and metabolic rate responses to *p*CO_2_ appear to be consistent across multiple natural systems characterised by *p*CO_2_ gradients such as those explored by Maas et al. (2012) and Lewis et al. (2013). However, recent work has suggested that marine invertebrates classed as *p*CO_2_ ‘sensitive’ may be those that experience trade-offs between energy metabolism and cellular homoeostasis under high *p*CO_2_ conditions. Homoeostatic capacity would be expected to be higher in ‘tolerant’ species, and thus they would not be expected to experience this trade-off (Turner et al. 2015).

In order to test the hypothesis that trade-offs between energy metabolism and cellular homoeostasis characterises the response of ‘sensitive’ species to high *p*CO_2_ conditions, as well as to examine the level of physiological adaptation or physiological plasticity to high *p*CO_2_ in the juvenile stages of a marine invertebrate assemblage, we carried out a series of in situ transplant experiments. These utilised juveniles of three species living inside and around the high *p*CO_2_ vent site of Castello Aragonese (Ischia, Naples, Italy). More specifically, we investigated the metabolic responses of the CO_2_ ‘tolerant’ *Dynamene bifida* Torelli, 1930 and CO_2_ ‘sensitive’ *Cymodoce truncata* (Leach, 1814) and *Dynamene torelliae* Holdich, 1968; ‘tolerant’ and ‘sensitive’ being defined by these species’ residence either inside or outside the vents, respectively (Kroeker et al. 2011; Ricevuto et al. 2012). Little is known about the effects of high *p*CO_2_ on early life stages of Peracarida, with only a few studies to date (Egilsdottir et al. 2009; Hauton et al. 2009). Juveniles of these three species are found on hard bottoms and also within *Posidonia oceanica* meadows and seaweed dominated habitats which at Ischia occur in both high and low *p*CO_2_ areas inside and outside the vent, respectively (Cigliano et al. 2010; Ricevuto et al. 2012). Our experimental design allowed us to compare and contrast the strategies used (i.e. degree of metabolic adaptation or metabolic acclimatisation) by this understudied, but ecologically important group of marine invertebrates with what we know about the annelid fauna found in the same area (Calosi et al. 2013b; Turner et al. 2015). The results obtained will allow us to unravel possible functional trade-offs among different traits and across different taxonomic groups, which may help explain the potential physiological sensitivity or the physiological tolerance of each of these species to high *p*CO_2_. After exposure to either low or high *p*CO2, we examined the concentration levels of fundamental aerobic and anaerobic metabolites (i.e. ATP, l-lactate, respectively) and those of carbonic anhydrase, an essential enzyme involved in an organism’s acid–base and respiratory function (Henry 1996) in each and every experimental individual (Bennett 1987; Calosi et al. 2013c; Turner et al. 2015). The use of an individual approach where we rigorously compared the levels of these three key metabolites allowed us to thoroughly test our hypothesis that marine invertebrates classed as *p*CO_2_ ‘sensitive’ may be those that experience trade-offs between energy metabolism and cellular homoeostasis under high *p*CO_2_ conditions.

## Materials and methods

### Animal collection and preparation for in situ transplant

All juvenile isopods were collected via SCUBA and snorkelling between June and September 2013. Juveniles of both *Dynamene* and *Cymodoce* are morphologically distinct from adults. Adult males are distinguished by the presence of a fully developed peraeonal bidentate process (*Dynamene*) or prominent pleonal tubercles (*Cymodoce*). Adult females of both genera feature modified mouthparts. Moreover, there is a sharp habitat separation between adults and juveniles of all three species used in this study. Adults (non-feeding) live in crevices, whereas juveniles (actively feeding) are found on seaweeds and seagrasses (M. Lorenti, personal observation).

Juveniles of the two ‘sensitive’ species *C. truncata* (*n* = 60, mean 13.07 ± 1.63 mg) and *D. torelliae* (*n* = 73, mean weight 3.06 ± 0.19 mg) were collected at two control sites (low *p*CO_2_/high pH) (C) at 1–2 m depth off St. Anna’s rocks, Ischia (Naples, Italy) (40°43′34″N, 13°57′35″E) approx. 600 m from the Castello south side vents, and at San Pietro promontory, Ischia, approximately 4 km from the venting site (40°44′48″N, 13°56′39″E) where pH values are representative of low *p*CO_2_ conditions (mean pH 8.13 ± 0.01) (Calosi et al. 2013b; Ricevuto et al. 2015), whilst juveniles of *D. bifida* (*n* = 90, mean weight 2.74 ± 0.21 mg) were collected at an acidified site (high *p*CO_2_/low pH) (A) 1–2 m depth on a rocky reef in an area with acidified conditions and high CO_2_ venting activity (>10 vents m^2^) on the south side of the Castello Aragonese, Ischia (40°43′53″N, 13°57′47″E) (mean pH 7.29 ± 0.04) (stations S2/S3 in previous studies, e.g. Cigliano et al. (2010)). For a detailed description of the collection and study areas see Calosi et al. (2013b). All specimens were transferred to the Villa Dohrn—Benthic Ecology Centre (approx. 4 km from the vents) within 30 min of collection using cool boxes filled with seawater of the appropriate pH from the collection site (approx. vol. = 10 L). The ‘cool box:isopod’ volume ensured that changes in temperature, salinity, O_2_ and pH were minimised during transport. Once in the laboratory, isopods were sorted from macroalgae by shaking the thalli inside plastic trashes, and maintained for 2 days prior to the experiment in glass bowls (approx. 20 indiv. per bowl), each containing 300 mL of natural seawater at the original pH/*p*CO_2_ which was changed daily. All glass bowls were kept in a temperature control room (*T* = 19 °C, 12 L:12 D cycle). Isopods were fed daily with fragments of macroalgae (mostly *Cladophora*, *Dictyota* and *Halopteris*) collected at the respective sampling sites.

### Experimental design, study area and experimental procedure

In order to characterise the presence of potential physiological mechanisms underpinning the apparent differences in sensitivity to high *p*CO_2_ of the three species of isopods investigated, as evidenced by their differential distribution around the CO_2_ vent, an in situ transplant experiment utilising the natural CO_2_ vents of Ischia was conducted. Isopods are highly motile compared to many other benthic marine invertebrate species. However, the juveniles collected for this study were all collected from macroalgae growing on rocky substrate and *P. oceanica* dead mat. *Cymodoce truncata* and *D. torelliae* collected from control areas (C) and were transplanted both to another control area (at the S. Pietro promontory) (e.g. CC) and to the acidified (vented) area (e.g. CA). In the case of *D. bifida*, individuals were collected from the acidified area (A) and transplanted to the control area (e.g. AC) and also maintained in the acidified area (e.g. AA). In each area, three stations were identified, approx. 50 m from each other and designated as C1, C2 and C3 for control, and A1, A2 and A3 for the high *p*CO_2_ vented area (c.f. Calosi et al. 2013b), in order to allow for spatial replication. Each station consisted of a weighted line with a buoy (approx. 2.5 m depth) to which the experimental containers or ‘transplantation chambers’ (TCs) could be attached. These were constructed from white PVC tubes (diameter = 4 cm, length = 11 cm) with a nytal plankton net (mesh = 100 µm) fixed to both ends. This net size allowed for the continual flow through of seawater, but at the same time prevented the isopods from escaping, being washed away or being predated upon.

On the day of deployment, isopods were transferred to the TCs (max. 15 indiv. *per* TC) underwater. These were then transferred to tanks (approx. vol. 10 L each, three TCs *per* tank) and transported to the experimental area: directly from land for the control area and via boat in the case of the acidified area. TCs were then immediately deployed by SCUBA to each station (*n* = 1 TC *per* station) in both the control and acidified areas where they remained for 5 days under natural conditions, according to the experimental design by Calosi et al. (2013b) and Turner et al. (2015). Seawater temperature, salinity and pH were measured at each station at the same time each day during the 5 days experimental period, and diel flux was minimal in the abiotic parameters measured (Lucey 2016; Lucey et al. 2016). Seawater samples were also taken for total alkalinity analyses (see Suppl. Mat.).

After 5 days exposure, TCs were recovered by SCUBA, placed underwater (in order to avoid any exposure to air) in 10-L tanks containing fresh seawater of the appropriate pH collected from the respective experimental areas. To minimise environmental shocks, isopods were transported to the laboratory within 30 min. Upon arrival in the laboratory, isopods were rapidly and carefully removed from the TCs, immediately weighed and snap frozen in liquid nitrogen inside 1.5-mL screw cap microcentrifuge tubes before being shipped to the Marine Biology and Ecology Research Centre (MBERC) at Plymouth University on dry ice. At MBERC, the concentration levels of fundamental aerobic and anaerobic metabolites were determined in each and every experimental individual (Bennett 1987; Calosi et al. 2013c; Turner et al. 2015). The use of an individual approach where we rigorously compared the levels of these three key metabolites allowed us to thoroughly test our hypothesis that marine invertebrates classed as *p*CO_2_ ‘sensitive’ may be those that experience trade-offs between energy metabolism and cellular homoeostasis under high *p*CO_2_ conditions. We decided to focus on two of the most fundamental indicators of metabolism: ATP and l-lactate. ATP is the primary source of free energy in all living cells, produced by *aerobic metabolism,* whereas l-lactate is the main indicator of *anaerobic metabolism* in crustaceans and thus can be indicative of metabolic stress (Urich 1994). We also measured levels of carbonic anhydrase, an essential enzyme involved in an organism’s acid–base and respiratory function (Henry 1996). Levels of this enzyme can also inform on biomineralisation processes, including in low-calcifying groups including Peracarida (Meyran et al. 1987). Concentrations of each of these were examined in each individual tested after exposure to either low or high *p*CO2. We hypothesised that when exposed to high *p*CO_2_ conditions, ‘sensitive’ species will upregulate their metabolic machinery to maintain homoeostasis (Calosi et al. 2013b) causing an associated increase in ATP (Turner et al. 2015). If these species have resorted to anaerobic respiration to maintain ATP production, then l-lactate concentrations will also increase. These ‘sensitive’ species would also need to upregulate acid–base and respiratory function upon exposure to high *p*CO2 conditions; therefore, levels of carbonic anhydrase would also be likely to increase. ‘Tolerant’ species exposed to low *p*CO_2_ conditions would likely experience less demand on their acid–base and metabolic function resulting in lower levels of ATP, l-lactate and carbonic anhydrase, the exact levels of which as well as the ratio between levels could indicate any metabolic trade-off response between different physiological processes as well as the degree of adaptation or acclimatisation of these species to high *p*CO_2_ conditions (Calosi et al. 2013b).

### Biochemistry

At the MBERC laboratory, specimen levels of ATP, l-lactate and carbonic anhydrase were determined. Beforehand whole animal tissue extracts were prepared. All extraction steps were completed in a temperature controlled room at 4 °C. Individuals were removed from storage at −80 °C and homogenised by hand using chilled disposable plastic micropestles (Eppendorf, Eppendorf AG, Hamburg, Germany) in 1.5-mL microcentrifuge tubes containing ice-cold 5 % trichloroacetic acid (TCA) at a ratio of 16:1 (TCA µL: body mass mg). The homogenate was then centrifuged for 10 min at 17,000*g* at 4 °C. The resulting supernatant was then removed to a second ice-chilled 1.5-mL microcentrifuge tube and used to determine [ATP], [l-lactate] and [carbonic anhydrase].

Concentrations of l-lactate and carbonic anhydrase were determined spectrophotometrically in the isopod extracts. These assays were undertaken in a microplate format using a VERSAmax™ plate reader (Molecular Devices, Sunnyvale, CA, USA). l-lactate concentrations were assayed using a commercial kit (l-lactate 735, Trinity Biotech Wicklow, Ireland). CA concentrations were measured using the methods detailed in Ivanina et al. (2013) but adapted to a microplate format.

ATP concentrations were determined using a commercial luciferase-based kit (ATP Kit SL 144-041, BioThema, Handen, Sweden). The reaction between luciferase and luciferin in the presence of ATP produces light with the amount of light emitted being directly proportional to the amount of ATP present. Luminescence was measured using a luminometer (Pi-102, Hygiena LLC, Camarillo, CA, USA). The difference in the slope of the reaction with and without the internal ATP standard was used to determine sample ATP concentration.

### Statistical analyses

GLM tests were used to investigate the effect of exposure to high or low *p*CO_2_ conditions found in the transplanted areas (inside or outside the CO_2_ vents, depending on species) on the mean cellular physiological traits investigated separately, with the term ‘station’ as a random factor nested within the *p*CO_2_ treatment and individual ‘body mass’ as a covariate. All data met assumptions for normality, although some data for carbonic anhydrase had to be log_10_ transformed beforehand (as indicated in Table 1) and for homogeneity of variances. In a preliminary analysis, both the terms ‘station’ and ‘body mass’ (within the range tested) were shown not to have a significant effect on the biological variables investigated and were therefore removed. To rigorously test our hypothesis whilst avoiding any limitation surrounding the use of the ‘golden mean’, individual approach analyses were also utilised (Bennett 1987), where individual values for the levels of the three key metabolites measured were correlated using the Pearson correlation test. All analyses were conducted in SPSS version 21.

**Table 1:** Mean ± SEM for [metabolite] and [carbonic anhydrase] in the isopods *Cymodoce truncata*, *Dynamene torelliae* and *Dynamene bifida* after exposure to control or acidified conditions

	Mean [control]	*n*	Mean [acidified]	*n*	*F*	df	*P*
‘Sensitive’ species							
*Cymodoce truncata*							
[ATP] (nmol mg^−1^)	1.95 ± 0.13	19	1.85 ± 0.18	15	0.220	1	0.642
[l-lactate] (nmol mg^−1^)	2.74 ± 0.18	18	2.18 ± 0.14	13	5.166	1	**0.031**
[Carbonic anhydrase] (U mg^−1^ protein)*	0.66 ± 0.14	12	0.27 ± 0.07	11	5.523	1	**0.029**
* Dynamene torelliae*							
[ATP] (nmol mg^−1^)*	1.71 ± 0.12	35	1.46 ± 0.13	19	1.531	1	0.222
[l-lactate] (nmol mg^−1^)	3.08 ± 0.18	20	2.38 ± 0.21	14	6.371	1	**0.017**
[Carbonic anhydrase] (U mg^−1^ protein)*	0.17 ± 0.05	25	0.43 ± 0.12	19	4.876	1	**0.033**
‘Tolerant’ species							
*Dynamene bifida*							
[ATP] (nmol mg^−1^)	1.60 ± 0.55	37	1.34 ± 0.07	30	4.759	1	**0.033**
[l-lactate] (nmol mg^−1^)	2.15 ± 0.18	22	2.51 ± 0.40	10	0.961	1	0.332
[Carbonic anhydrase] (U mg^−1^ protein)*	0.15 ± 0.02	30	0.17 ± 0.03	15	0.906	1	0.349

Results for the GLM tests are reported, with number of specimens investigated (*n*), F-ratio (*F*), degrees of freedom (df) and probability (*P*)

* log_10_ transformed prior to analysis

Significant *P* values are given in bold

## Results

Survival rates were overall good and comparable across all species investigated at the different *p*CO_2_ treatments, with an overall average survival rate of 74 % (Table S1). Results for the carbonate system (Table S2) confirmed that pH and *p*CO_2_ differed significantly outside (C) and inside (A) the CO_2_ vent areas, consistent with previous studies (Kroeker et al. 2011; Calosi et al. 2013b; Ricevuto et al. 2015).

**Fig. 1:**
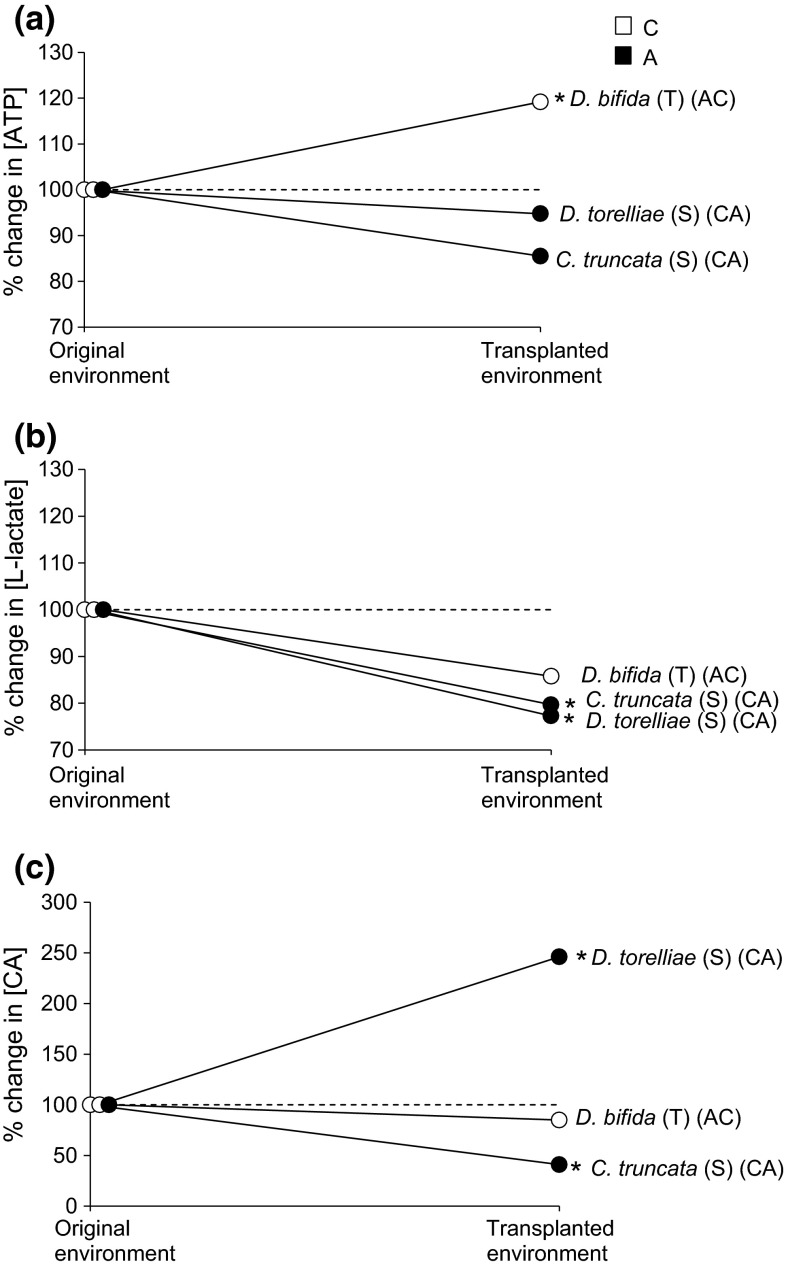
Reaction norms as contrast plots showing percentage change of mean **a** [ATP] (nmol mg^−1^), **b** [l-lactate] (nmol mg^−1^) and **c** [carbonic anhydrase (CA)] (nmol mg^−1^) in the isopods *Cymodoce truncata* [sensitive (S)], *Dynamene torelliae* [sensitive (S)] and *Dynamene bifida* [tolerant (T)] when isopods were exposed to high *p*CO_2_/low pH or low *p*CO_2_/high pH conditions in a transplanted environment either inside or outside the vented area. The sensitive *Cymodoce truncata* and *Dynamene torelliae* were collected from low *p*CO_2_/high pH conditions outside the vents (*C*) and transplanted to high *p*CO_2_/low pH conditions inside the vents (*A*) (e.g. *CA*). The tolerant *Dynamene bifida* were collected from high *p*CO_2_/low pH conditions within the vents (*A*) and transplanted to low *p*CO_2_/high pH conditions outside the vents (*C*) (e.g. *AC*). Mean biochemical parameter measured in original environment was set as 100 %, and mean biochemical parameter measured in transplanted environment recalculated accordingly. *Dashed line* indicates no change in reaction norm from 100 %. *Asterisk* indicates the presence of a significant difference between the mean biochemical parameter measured in the original environment and transplanted environment according to the GLM test (*P* < 0.05)

Following in situ transplantation inside TCs, the average survival rate of juvenile isopods was 74 %. Mean values for the biochemical parameters measured are given in Table 1. Exposure in situ of both the ‘sensitive’ *C. trunctata* and *D. torelliae* to high *p*CO_2_ did not exert a significant effect on [ATP] (Fig. 1a), whilst it leads to a significant decrease in mean [l-lactate] (Fig. 1b). In addition, [carbonic anhydrase] decreased significantly in *C. truncata* and increased significantly in *D. torelliae* (Fig. 1c) following exposure to high CO_2_. The ‘tolerant’ species *D. bifida* when transplanted from high *p*CO_2_ conditions to low *p*CO_2_ (AC) conditions showed a significant increase in [ATP] (Fig. 1a), and no significant difference in either [l-lactate] or [carbonic anhydrase] (Fig. 1b, c). Individual analyses revealed non-significant correlations between [ATP] and [l-lactate] and [ATP] and [carbonic anhydrase] for all three species examined. The exception was that of a positive relationship between [ATP] and [l-lactate] for the ‘sensitive’ *D. torelliae* exposed to low *p*CO_2_ (*r* = 0.482, *P* = 0.031). This relationship broke down when the isopods were exposed to high *p*CO_2_ conditions (*r* = 0.17, *P* = 0.561) (see Suppl. Mat.).

## Discussion

In this study, we show the differing metabolic responses of three closely related marine isopods to high and low *p*CO_2_ conditions during an in situ transplant experiment at a natural shallow water high *p*CO_2_ vent system. Our data demonstrate that differences in the distribution of these three species in and around the Ischia high *p*CO_2_ vent can begin to be explained by either the physiological adaptation or plasticity (acclimatisation response) of each species to high *p*CO_2_ conditions. In a broader context, our findings strengthen the idea that the distribution of marine invertebrates to future high *p*CO_2_ conditions will be, at least in part, dictated by their ability to increase their metabolic and homoeostatic capacities (Melzner et al. 2009; Calosi et al. 2013b; De Wit et al. 2015; Magozzi and Calosi 2015; Turner et al. 2015).

For the two sensitive species *C. truncata* and *D. torelliae,* there was no change in ATP concentration when they were exposed to high *p*CO_2_ conditions, with this being accompanied by a decrease in l-lactate concentration. This suggests that despite being considered sensitive to high *p*CO_2_, both these species have a degree of capacity for metabolic plasticity, at least during our short-term experiment, enabling them to maintain a certain level of homoeostasis when facing high *p*CO_2_ conditions, e.g. ATP production appears to be maintained without recourse to anaerobic pathways (Urich 1994). Some species of marine isopods have been recorded as being metabolically tolerant to extreme environmental conditions. The Baltic isopod *Saduria entomon* which is closely related to *Dynamene* and *Cymodoce* is able to maintain aerobic metabolism during conditions of extreme hypoxia (Hagerman and Vismann 1997) and several other isopod species, e.g. *Stenasellus virei* are known to have a high capacity for glyconeogenesis from lactate and a faster and more complete replenishment rate of ATP and arginine phosphate after hypoxic exposure (Hervant et al. 1997). *Saduria entomon* also appears to be metabolically tolerant to OA conditions (Jakubowska et al. 2013), whereas others are negatively impacted in terms of their metabolic (Alenius and Munguia 2012) and immunological (Wood et al. 2014) homoeostatic capacity. Our results suggest that metabolic capabilities enabling species to tolerate extreme conditions may be more widespread throughout isopods than previously thought, although longer exposure experiments are required to allow further exploration of this hypothesis. However, even though our results indicate that both *C. truncata* and *D. torelliae* possess the metabolic capability to withstand the high *p*CO_2_ conditions inside the vented area, there may still be other, as yet unknown, ecological factors, e.g. low food supply and/or limitations on these species interactions with the dominant macroalgae (Kroeker et al. 2011) that prevent these species inhabiting this site.

However, for the two sensitive species (*C. truncata* and *D. torelliae*), there is a species-specific difference in the reaction norm for carbonic anhydrase after exposure to high *p*CO_2_ conditions. In the case of *C. truncata,* there is a significant decrease in carbonic anhydrase concentration under high *p*CO_2_ conditions, whereas for *D. torelliae,* there is a significant increase. This evidence, together with the fact that in both species ATP production is maintained under high *p*CO_2_ conditions indicates the presence of physiological trade-offs. The utilisation of physiological trade-offs allowing the maintenance of energy metabolism at the expense of cellular homoeostasis has recently been proposed for another high *p*CO_2_ sensitive marine ectotherm, the fan worm, *Sabella spallanzanii* (Turner et al. 2015). This species is also found around the high *p*CO_2_ vent at Ischia but never inside the CO_2_ venting areas, and when exposed to high *p*CO_2_ conditions has been shown to upregulate ATP production at the apparent cost of carbonic anhydrase concentration (Turner et al. 2015). The results of the current study suggest that this physiological trade-off could also be present in *C. truncata*, thus suggesting that this strategy could be utilised by multiple taxa to maintain homoeostasis when facing high *p*CO_2_ conditions, at least in the short term. However, in the isopod species examined this mechanism remains less well defined when compared to that of the polychaete *S. spallanzanii.* Furthermore, even though a high *p*CO_2_ induced downregulation of carbonic anhydrase has also previously been shown in other low-calcifying marine invertebrate species, such as the crab *Carcinus maenas* (Fehsenfeld et al. 2011), it is unlikely that this represents a long-term adaptive strategy. Instead this physiological plastic response may have evolved to extend aerobic scope under short-term exposure to high *p*CO_2_ conditions. This could be particularly relevant for groups such as isopods. These have a fairly low degree of calcification and are known to be able to successfully regulate internal acid–base balance. However, we know little about any effect of long-term exposure to high *p*CO_2_ conditions on acid–base regulation or the metabolic costs of maintaining homoeostasis (c.f. Wood et al. 2008; Seibel et al. 2012) which could, at least for some species be problematic. The isopod cuticle contains magnesium calcite which is highly soluble. Long-term exposure to OA-associated changes to oceanic carbonate chemistry could result in surface sea water becoming undersaturated with respect to this mineral (Feely et al. 2004; Andersson et al. 2008; Neues and Epple 2008). Therefore, the fact that it is likely that any long-term downregulation in carbonic anhydrase could ultimately result in respiratory function and acid–base processes being compromised (Henry 1996) means that this strategy is unlikely to be sustainable in the long term. This may be most true as organisms also undertake energy demanding processes, such as growth and reproduction. In contrast, in the current study there is a significant increase in carbonic anhydrase for the sensitive *D. torelliae* after exposure to high *p*CO_2_ conditions. This is consistent with what has previously been recorded for other marine calcifiers such as the bivalves *Crassostrea virginica* and *Mercenaria mercenaria*, for which exposure to high *p*CO_2_ has been shown to cause an increase in carbonic anhydrase concentration to maintain CaCO_3_ deposition (Ivanina et al. 2013). Whilst the role of carbonic anhydrase in shell calcification is fundamental for calcifying species, in both calcifying and non-calcifying marine invertebrates, this increase in carbonic anhydrase concentration is needed to ultimately maintain cellular homoeostasis through facilitating gas exchange and CO_2_ excretion. When these CO_2_ sensitive isopod species are faced with high *p*CO_2_ conditions it appears they are utilising the up or down regulation of carbonic anhydrase, as a physiological trade-off to ultimately extend their metabolic capacity. This also suggests that despite being CO_2_ sensitive, there may be differing degrees of sensitivity between closely related taxa with a species-specific degree of metabolic resilience being potentially advantageous to enable them to cope with future OA conditions. Furthermore, the identification of these kinds of alternative physiological strategies used by marine invertebrates when they are exposed to high *p*CO_2_ conditions may have important implications for our understanding of the generality of the physiological mechanisms involved in species responses to OA (Melzner et al. 2009; Stillman and Paganini 2015).

*Dynamene bifida* responds with a different metabolic fingerprint to the exposure to high and low *p*CO_2_ conditions. This species is known to thrive inside the high *p*CO_2_ vented area (Ricevuto et al. 2012) leading to the classification of this species as CO_2_ tolerant. *Dynamene bifida* significantly increases ATP production when transplanted from high to low *p*CO_2_ conditions, this being an approximately 20 % increase compared to individuals of *D. bifida* collected from and maintained inside the vents. This is a similar situation to that seen in the CO_2_ tolerant annelid species *Platynereis massiliensis* which is also found in large numbers inside the high *p*CO_2_ vent at Ischia (Lucey et al. 2015). When individuals of *P. massiliensis* are transplanted from high to low *p*CO_2_ conditions, this species also elevates its metabolic rate compared to that of individuals maintained inside the vented high *p*CO_2_ area (Calosi et al. 2013b). This suggests that this could be a multi-taxa response whereby the metabolism of vent individuals is maintained at high levels, compared to those that inhabit low *p*CO_2_ areas (Pörtner and Farrell 2008; Calosi et al. 2013b; Melzner et al. 2013). Furthermore, the fact that this increase in ATP production in *D. bifida* when transported to low *p*CO_2_ conditions is also not accompanied by a significant difference in l-lactate production could suggest that this species has the capacity to upregulate its metabolism without incurring the upregulation of anaerobic pathways. Overall, this could suggest that these species have metabolically adapted to high *p*CO_2_ conditions at the Ischia vent site.

The tolerant species, *D. bifida* is found throughout the Mediterranean (Torelli 1930; Ledoyer 1962; Holdich 1970; van der Land 2001; Kirkim et al. 2006; Koukouras 2010) in low CO_2_ conditions. Both a change in food quality (Garrard et al. 2014) and the nature of plant infochemicals (Zupo et al. 2015) have been cited as habitat attributes that can influence the distribution of isopods under ocean acidification conditions. However, it is possible that for *D. bifida* there are also ecological advantages, such as protection from predation and/or increased availability of food (Kroeker et al. 2011) for invading the high *p*CO_2_ vented area at Ischia.

The results of our study suggest that the distribution of the sphaeromatid isopod assemblage around the high *p*CO_2_ vent at Ischia may be explained, at least in part, by their ability to acclimatise or adapt to the high *p*CO_2_ conditions. However, other factors may also influence the distribution of these species. The life cycles of *Cymodoce* and *Dynamene* and the behaviour of juveniles are characterised on a diel basis, by habitat shifts which probably rely on a complex of environmental signals (e.g. Sanchez-Jerez et al. 1999). Previously, CO_2_ concentration has been shown to influence sphaeromatid behaviour (Alenius and Munguia 2012), including diel vertical migrations in vegetated biotopes (Dumay 1971) suggesting there may be, as yet further complex interactions of physiology and behaviour that may also influence the distribution of individual species around the high *p*CO_2_ vents at Ischia. Nonetheless, the ability of organisms to adjust their physiology to maintain metabolic homoeostasis via either adaptation or acclimatisation in response to *p*CO_2_ will influence the distribution of species around areas of high *p*CO_2_ conditions. Ultimately this can have genetic, ecological and conservation implications, which are particularly important when predictions are made about how marine life will respond to future global change scenarios, e.g. OA. Our results highlight how the importance of understanding the physiological responses of marine species to OA can assist us in making predictions about how marine communities may respond to this future environmental challenge. Physiological information can be used to promote active conservation exercises (Wikelski and Cooke 2006) and help identify priorities for the preservation of marine biodiversity and ecosystems function (Seijo et al. 2016).

## Electronic supplementary material

Below is the link to the electronic supplementary material.

Supplementary material 1 (PDF 193 kb)
