# Supplementary material for: Metabolic responses to high pCO2 conditions at a CO2 vent site in juveniles of a marine isopod species assemblage
Source: Mar Biol. 2016 Sep 20;163(10):211. doi: 10.1007/s00227-016-2984-x (PMC5030223; doi:10.1007/s00227-016-2984-x)
Supplement: Supplementary file 1 — Supplementary material 1 (PDF 193 kb) [file 227_2016_2984_MOESM1_ESM.pdf]

Supplementary materials for 'Metabolic responses to high pCO_2 conditions at a CO_2 vent site in juveniles of a marine isopod species assemblage'

Lucy M. Turner, Elena Ricevuto, Alexia Massa Gallucci, Maurizio Lorenti, Maria-Cristina Gambi, Piero Calosi

Additional details for Materials and Methods

Environmental monitoring and profiles

Seawater temperature, salinity and pH were measured at each station daily during the 5 d experimental period using a digital thermometer with thermocouple (HH806AU, Omega Ltd., Manchester, UK), a refractometer (TA 197 LFMulti350, WTW, Weilheim, Germany) and a pH microelectrode (Seven Easy pH InLab, Mettler-Toledo Ltd., Beaumont Leys, UK), maintained at ambient seawater temperature, coupled to a pH meter (Sevengo, Mettler-Toledo Ltd.), calibrated using pH standards (pH 4.01, 7.00, 9.21 at 25°C, Mettler-Toledo Ltd.) and also maintained at ambient seawater temperature. To determine sea water total alkalinity (TA), samples of sea water (vol. = 100 mL) were also collected at each station daily using Kevlar bottles with a secure tight lid. These were transported inside a cool box to the laboratory and poisoned upon arrival with $HgCl_2$ within approximately 1 h from collection. Samples were subsequently shipped to the laboratory of the Marine Biology and Ecology Research Centre (MBERC) (Plymouth, UK) where TA was determined using an alkalinity titrator (AS-ALK2, Apollo SciTech, Bogart, USA).

Seawater dissolved inorganic carbon (DIC), partial pressure of CO_2 (pCO_2), calcite and aragonite saturation (Ω_{calc} and Ω_{ara}), bicarbonate and carbonate ion concentration ([HCO_3^-] and [CO_3^{2-}], respectively) were calculated from pH and TA measurements using the software program CO2SYS (Pierrot, 2006) with dissociation constants from (Mehrbach, 1973) refit by (Dickson, 1987) and [KSO_4] using (Dickson, 1990).

Additional details for Results

Table S1. Survival rates for the isopods *Cymodoce truncata*, *Dynamene torelliae* and *Dynamene bifida* after exposure to control or acidified conditions.

	Treatment	Station	% survival
**'Sensitive' species**			
Cymodoce truncata	C-C	C1	0
		C2	100.0
		C3	90.0
	C-A	A4	40.0
		A5	70.0
		A6	50.0
Dynamene torelliae	C-C	C1	100.0
		C2	100.0
		C3	100.0
	C-A	A4	16.67
		A5	83.33
		A6	75.0
**'Tolerant' species**			
Dynamene bifida	A-A	A4	73.33
		A5	53.33
		A6	100.0
	A-C	C1	86.67
		C2	86.67
		C3	100.0

Table S2. Values (mean ± SEM) for physico-chemical parameters of the seawater used when exposing isopods to: (i) current pCO_2/pH conditions (‘control’ stations C1, C2 and C3) and (ii) elevated pCO_2/low pH conditions (‘acidified’ stations A1, A2, A3). Salinity, temperature, pH_{NBS} (Mettler-Toledo pH meter, Beaumont Leys, UK), total alkalinity (TA) (AS-ALK2, Apollo SciTech, Bogart, USA), dissolved inorganic carbon (DIC), carbon dioxide partial pressure (pCO_2), bicarbonate and carbonate ion concentration ($[HCO_3^-]$ and $[CO_3^{2-}]$), calcite and aragonite saturation state (Ωcal and Ωara) are provided. The significant difference in temperature between the two sites was negligible and within the range of natural fluctuation experienced at these sites. Preliminary statistical analyses showed no effect of temperature differences on the biochemical parameters investigated (maximum $F_{5,\,34}$ = 1.803, P = 0.139).

**Parameter**	**Control**				**Acidified**			
	**C1**	**C2**	**C3**	**Overall**	**A1**	**A2**	**A3**	**overall**
**Salinity**	36.93 ± 0.11	37.00 ± 0.10	37.02 ± 0.09	36.98 ± 0.06	37.00 ± 0.08	37.08 ± 0.09	37.05 ± 0.12	37.04 ± 0.06
**Temperature (°C)**	21.29 ± 0.30^{A}	21.36 ± 0.32^{A}	21.37 ± 0.31^{A}	21.34 ± 0.18^{a}	20.17 ± 0.18^{B}	20.12 ± 0.18^{B}	20.41 ± 0.16^{B}	20.23 ± 0.10^{b}
**pH**	8.12 ± 0.01^{A}	8.13 ± 0.01^{A}	8.15 ± 0.01^{A}	8.13 ± 0.01^{a}	7.49 ± 0.06^{B}	7.16 ± 0.08^{C}	7.21 ± 0.06^{C}	7.29 ± 0.04^{b}
**TA (μequiv kg^{-1})**	2612.93 ± 4.68	2589.79 ± 6.92	2600.38 ± 4.05	2601.33 ± 3.21	2620.00 ± 4.19	2597.40 ± 11.21	2606.72 ± 14.29	2608.15 ± 6.22
**DIC (μmol kg^{-1})***	2333.39 ± 9.19^{A}	2305.76 ± 13.71^{A}	2305.98 ± 9.62^{A}	2315.40 ± 6.39^{a}	2640.00 ± 31.65^{B}	2802.89 ± 47.34^{C}	2759.76 ± 41.80^{C}	2733.52 ± 24.25^{b}
***p*CO2 (μatm)***	518.05 ± 13.50^{A}	505.30 ± 19.00^{A}	482.89 ± 14.19^{A}	502.24 ± 9.05^{a}	3682.36 ± 702.65^{B}	8608.77 ± 1150.84^{C}	6216.07 ± 798.54^{D}	6144.17 ± 552.63^{b}
**$[HCO_3^-]$ (μmol kg^{-1})***	2109.27 ± 12.82^{A}	2079.87 ± 18.26^{A}	2073.80 ± 13.79^{A}	2088.02 ± 8.73^{a}	2455.28 ± 20.62^{B}	2481.77 ± 27.81^{B}	2509.83 ± 23.58^{B}	2482.30 ± 13.96^{b}
**$[CO_3^{2-}]$ (μmol kg^{-1})***	208.07 ± 4.54^{A}	210.26 ± 5.61^{A}	217.29 ± 4.98^{A}	211.84 ± 2.90^{a}	68.08 ± 7.79^{B}	47.76 ± 11.14^{C}	40.02 ± 6.50^{C}	52.00 ± 5.08^{b}
**Ω^{b}cal**	4.91 ± 0.11^{A}	4.96 ± 0.13^{A}	5.12 ± 0.12^{A}	5.00 ± 0.07^{a}	1.60 ± 0.18^{B}	1.12 ± 0.26^{C}	0.94 ± 0.15^{C}	1.22 ± 0.12^{b}
**Ω^{b}ara**	3.21 ± 0.07^{A}	3.24 ± 0.09^{A}	3.35 ± 0.08^{A}	3.27 ± 0.05^{a}	1.05 ± 0.12^{B}	0.73 ± 0.17^{C}	0.61 ± 0.10^{C}	0.80 ± 0.08^{b}

Different capital letters (A,B,C,D) indicate significant differences in parameter values from different stations.
Different lowercase letters (a,b) indicate significant differences in parameter values from different treatments.
[*]Parameters that were calculated using the CO2SYS program (Pierrot et al., 2006), using the dissociation constants of Mehrbach et al. (1973) as refitted by Dickson & Millero (1987) and [KSO4] using Dickson (1990).

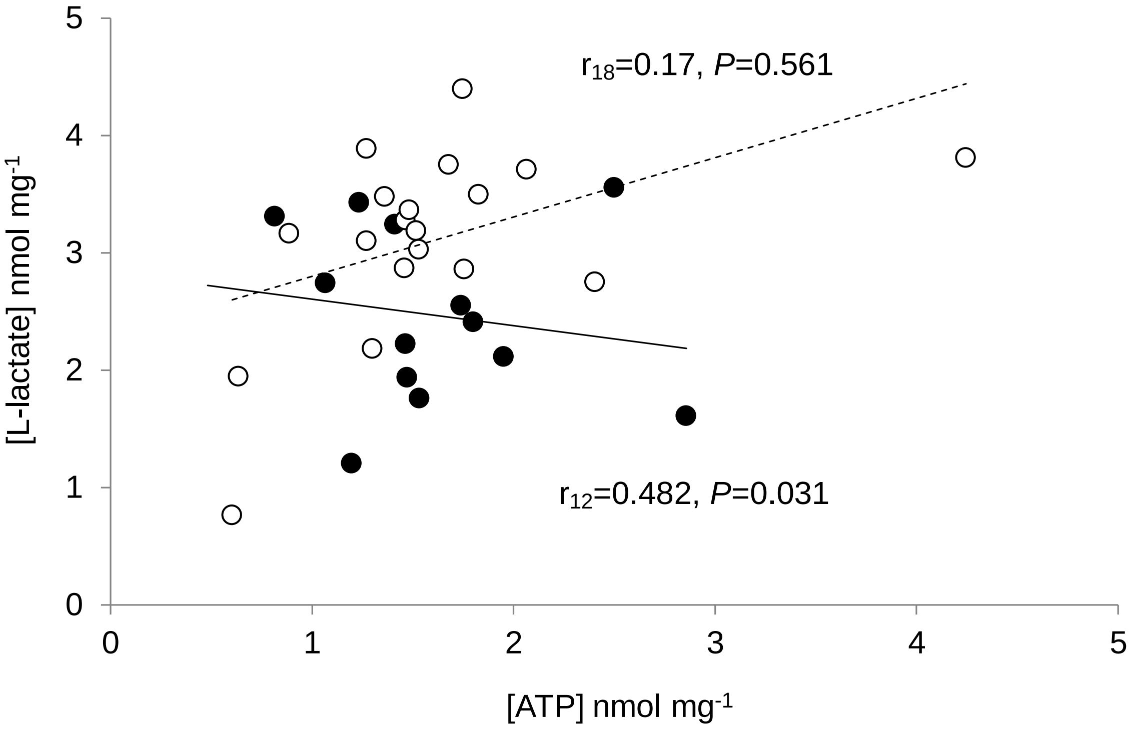

Fig. S1 The relationship between individuals' levels of ATP (nmol mg^{-1}) and L-lactate (nmol mg^{-1}) in *Dynamene torelliae* when exposed to high pCO_2 (filled circles) or low pCO_2 (open circles) conditions. Data points represent individual measurements. The Pearson correlation coefficient and degrees of freedom, together with the probability values (*P*) are provided. Regression lines are shown as solid or dashed lines denoting significant and non-significant relationships, respectively.

References

Dickson AG (1990) Thermodynamics of the dissociation of boric acid in synthetic seawater from 273.15 to 318.15 K. Deep Sea Res 37:755–766

Dickson AG, Millero FJ (1987) A comparison of the equilibrium constants for the dissociation of carbonic acid in seawater media. Deep Sea Res 34:1733–1743

Mehrbach C, Culberson CH, Hawley JE, Pytkowicz RM (1973) Measurement of the apparent dissociation constants of carbonic acid in seawater at atmospheric pressure. Limnol Oceanogr 18:897-907

Pierrot D, Lewis E, Wallace DWR (2006) MS Excel program developed for CO_2 system calculations, ORNL/CDIAC-105. Oak Ridge, TN: Carbon Dioxide Information Analysis Center, Oak Ridge National Laboratory, U.S. Department of Energy
